# Distinct Clinical Impact and Biological Function of Angiopoietin and Angiopoietin-like Proteins in Human Breast Cancer

**DOI:** 10.3390/cells10102590

**Published:** 2021-09-29

**Authors:** Hui Yang, Melody Zhang, Xuan-Yu Mao, Hang Chang, Jesus Perez-Losada, Jian-Hua Mao

**Affiliations:** 1 Biological Systems and Engineering Division, Lawrence Berkeley National Laboratory, Berkeley, CA 94720, USA; huiyang@lbl.gov (H.Y.); melodyzhang00@g.ucla.edu (M.Z.); s_mao@u.pacific.edu (X.-Y.M.); hchang@lbl.gov (H.C.); 2 Hubei Key Laboratory of Tumor Biological Behaviors, Department of Radiation and Medical Oncology, Hubei Cancer Clinical Study Centre, Zhongnan Hospital of Wuhan University, Wuhan 430071, China; 3 Undergraduate Program at Department of Ecology and Evolutionary Biology, University of California Los Angeles, Los Angeles, CA 90095, USA; 4 Instituto de Biología Molecular y Celular del Cáncer (CIC-IBMCC), Universidad de Salamanca/CSIC, 37007 Salamanca, Spain; jperezlosada@usal.es; 5 Instituto de Investigación Biomédica de Salamanca (IBSAL), 37007 Salamanca, Spain

**Keywords:** integrated omics, breast cancer, angiopoietin protein, angiopoietin-like protein, prognosis

## Abstract

Secreted angiopoietin/angiopoietin-like (*ANGPT*/*ANGPTL*) proteins are involved in many biological processes. However, the role of these proteins in human breast cancers (BCs) remains largely unclear. Here, we conducted integrated omics analyses to evaluate the clinical impact of *ANGPT*/*ANGPTL* proteins and to elucidate their biological functions. In BCs, we identified rare mutations in *ANGPT*/*ANGPTL* genes, frequent gains of *ANGPT1*, *ANGPT4*, and *ANGPTL1*, and frequent losses of *ANGPT2*, *ANGPTL5*, and *ANGPTL7*, but observed that *ANGPTL1*, *2*, and *4* were robustly downregulated in multiple datasets. The expression levels of *ANGPTL1*, *5*, and *8* were positively correlated with overall survival (OS), while the expression levels of *ANGPTL4* were negatively correlated with OS. Additionally, the expression levels of *ANGPTL1* and *7* were positively correlated with distant metastasis-free survival (DMFS), while the expression levels of *ANGPT2* and *ANGPTL4* were negatively correlated with DMFS. The prognostic impacts of *ANGPT*/*ANGPTL* genes depended on the molecular subtypes and on clinical factors. We discovered that various *ANGPT*/*ANGPTL* genes were co-expressed with various genes involved in different pathways. Finally, with the exception of *ANGPTL3*, the remaining genes showed significant correlations with cancer-associated fibroblasts, endothelial cells, and microenvironment score, whereas only *ANGPTL6* was significantly correlated with immune score. Our findings provide strong evidence for the distinct clinical impact and biological function of *ANGPT*/*ANGPTL* proteins, but the question of whether some of them could be potential therapeutic targets still needs further investigation in BCs.

## 1. Introduction

Breast cancer (BC) is one of the leading causes of death among women worldwide [1,2,3]. It is well known that BC is a complex and heterogeneous disease with substantial variation in its molecular and clinical characteristics [4,5]. Multi-omics technologies have proved to be invaluable tools for deconvoluting the heterogeneity and complexity of somatic BC genetics, providing a tremendous amount of information relating to the definition of new biomarkers for diagnosis, prognosis, and the prediction of therapeutic response and to the identification of new potential therapeutic targets. Based on these findings, a few genomic prognostic tests are available for BC, such as Oncotype Dx (Genomic Health Inc., Redwood City, CA, USA) and MammaPrint (Agendia, Amsterdam, The Netherlands). However, while some improvements have been made in the diagnosis and treatment of BC, the prognosis for, and the survival of, patients with metastatic cancer have not dramatically changed. The demand for precision cancer medicine has never been higher, and therefore, it is critical to identify new potential therapeutic targets.

Angiogenesis is one of the hallmarks of human cancers. Tumors require sufficient vasculature to grow beyond a certain size, invade nearby tissue, or spread throughout the body [6]. To initiate tumor angiogenesis, tumor cells release molecules that send signals to surrounding normal host tissue. These signals activate specific genes in the host tissue to stimulate the growth of new vasculature towards the tumor [7]. Many cellular and molecular mechanisms involved in tumor angiogenesis have been well documented, for example, vascular endothelium growth factors and their receptors are key factors in regulating endothelial cell proliferation and migration to form the basis of any vessel [8]. The effective inhibition of tumor angiogenesis can reduce or slow down the spread and growth of some types of cancer. Several angiogenesis inhibitors have been approved by the U.S. Food and Drug Administration (FDA) for treating cancer [9,10].

Secreted angiopoietin/angiopoietin-like (ANGPT/ANGPTL) proteins regulate angiogenesis and ensure vascular integrity and permeability [11,12,13]. There are three angiopoietin proteins (ANGPT1, ANGPT2, and ANGPT4) and eight angiopoietin-like proteins (ANGPTL1-8). Increasing evidence has shown that some of these genes play an important role in tumor development and progression [14,15]. For example, a few studies have demonstrated that ANGPTL1 functions as a tumor suppressor gene in breast cancer [16], hepatocellular carcinoma [17,18], colorectal cancer [19,20,21], thyroid cancer [22], and lung cancer [16]. However, the role of these proteins in human BCs remains largely unknown. In this study, we used multiple bioinformatics tools to evaluate the clinical impact of the ANGPT/ANGPTL proteins and elucidate their biological functions in BCs. Gaining an insight into understanding *ANGPT*/*ANGPTL* genes is essential for developing a promising strategy for diagnosing and treating human cancers.

## 2. Materials and Methods

The mutational frequency and DNA copy number changes of *ANGPT*/*ANGPTL* genes were obtained with respect to invasive breast carcinomas via cBioPortal (http://www.cbioportal.org/) from the Cancer Genome Atlas (TCGA-BRCA, PanCancer Atlas) database on 1 October 2020 [23,24]. The Spearman correlation between the gene DNA copy number and the expression in the TCGA-BRCA database was calculated using SPSS (IBM SPSS statistics version 24). The Catalogue of Somatic Mutations in Cancer (COSMIC, v92) database (https://cancer.sanger.ac.uk/cosmic) was used to verify the mutational frequencies on 1 October 2020 [25].

Gene transcript data for normal and tumor tissues were downloaded from the National Center for Biotechnology Information (NCBI) Gene Expression Omnibus (GEO) (GSE3744, GSE10780, GSE21422, and GSE29044). The fold change (FC) and the significance were calculated for each gene using GEO2R (|log_2_(FC)| > 1.5 and adjusted *p*-values < 0.05). Further comparisons of gene expression data between normal, cancer-adjacent, and cancer tissues in the Cancer Genome Atlas (TCGA) were performed using Breast Cancer Gene-Expression Miner v4.6 (bc-GenExMiner v4.6, http://bcgenex.ico.unicancer.fr/BC-GEM/GEM-Accueil.php?js=1) from 1 October 2020 [26,27,28].

We performed a meta-analysis of the association between *ANGPT*/*ANGPTL* genes and the overall survival (OS) and distant metastasis-free survival (DMFS), generated Kaplan–Meier survival curve plots by dividing the gene expressions into tertiles, and identified the genes co-expressed with *ANGPT*/*ANGPTL* genes in RNA-Seq data with criteria |r| ≥ 0.40 and *p* < 1.00 x10^−4^ using bc-GenExMiner v4.6. Gene Ontology (GO) and Kyoto Encyclopedia of Genes and Genomes (KEGG) pathway enrichment analyses were also performed (clusterProfiler package in R, Version 3.16.1).

We searched all possible datasets and only found three datasets (GSE96058, METABRIC, and TCGA) that contained both the transcriptional data of all *ANGPT*/*ANGPTL* genes and clinical information. The GSE96058 dataset was downloaded from the Gene Expression Omnibus (GEO) database, while the METABRIC and TCGA datasets were downloaded from cBioPortal. Univariate and multivariate Cox regression analyses were executed in these three datasets using SPSS.

The tumor immune infiltration scores, stroma scores, microenvironment scores, cancer-associated fibroblasts, and endothelial cells in TCGA were downloaded from TIMER2.0 (http://timer.cistrome.org/) on 1 October 2020 [29], and were enumerated from transcriptomes using the xCell method, a novel gene-signature-based method [30]. The Spearman correlations between the expression of *ANGPT*/*ANGPTL* genes and these biological factors in the TCGA-BRCA data were calculated using SPSS (IBM SPSS statistics version 24).

## 3. Results

### 3.1. Genomic Alterations in ANGPT/ANGPTL Genes in Breast Cancers

To gain insight into understanding the role of *ANGPT*/*ANGPTL* genes in human BC development and progression, we first investigated their genomic alterations in BCs. Upon mining the TCGA-BRCA data, we observed low mutational frequency without hotspots in *ANGPT*/*ANGPTL* genes. These observations were further verified by the frequencies reported in the COSMIC database (Table 1), indicating that *ANGPT*/*ANGPTL* genes are rarely mutated in BCs.

**Figure 1:**
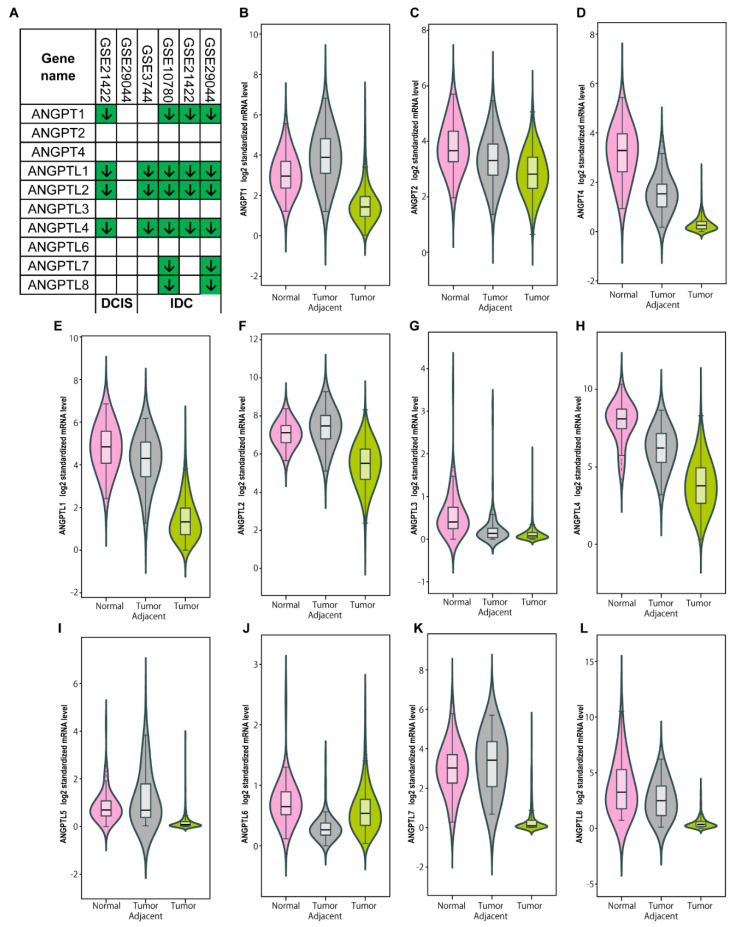
*ANGPTL1*, *2*, and *4* are consistently downregulated in breast cancers (BCs). (**A**) Comparison of transcriptional expression of *ANGPT*/*ANGPTL* genes between normal breast and BC tissues in multiple microarray datasets. Significantly decreased gene expression (1.5-fold; adjusted *p* < 0.05) is shown in green with an arrow. (**B**–**L**) Box plot of transcriptional expression of the *ANGPT*/*ANGPTL* genes in normal, tumor-adjacent, and tumor tissues by RNA-seq analysis in TCGA dataset. Boxes represent the median and interquartile ranges between the first and third quartiles. Number of normal breast tissues = 92; number of breast-tumor-adjacent tissues = 104; number of breast tumor tissues = 1034.

**Figure 2:**
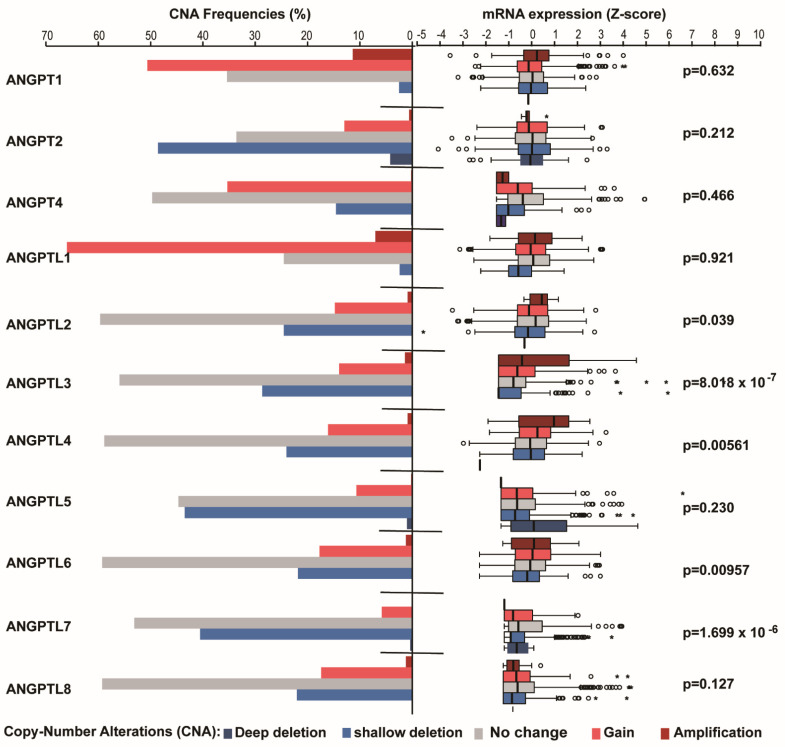
Correlation between DNA copy number of *ANGPT*/*ANGPTL* genes and their transcriptional expression in TCGA-BRCA. Left panel: frequency of DNA copy number alteration (CNA) in *ANGPT*/*ANGPTL* genes. Right panel: box plot of the relationship between DNA copy number and gene expression for *ANGPT*/*ANGPTL* genes in BCs. Stars indicate extreme outliers while circles indicate mild outliers. The *p*-values were obtained from Spearman correlation analysis between gene DNA copy number and expression.

Next, we investigated the changes in the transcriptional levels of *ANGPT*/*ANGPTL* genes by comparing their expression profiles in normal breast and BC tissues using GEO2R. We observed that *ANGPTL1*, *2*, and *4* were robustly and significantly downregulated in invasive ductal carcinoma (IDC) across all microarray datasets in the GEO database (Figure 1A, Table S1). However, we discovered that the transcriptional levels of all *ANGPT*/*ANGPTL* genes were significantly lower in BCs than in normal breast tissues in the TCGA dataset (Figure 1B–L). Moreover, the significant downregulation of *ANGPT2*, *ANGPT2*, *ANGPTL1*, *ANGPTL4*, and *ANGPTL6* was found in tumor-adjacent tissues (Figure 1B–L). Interestingly, the downregulation of *ANGPT1* and *ANGPTL1*, *2*, and *4* was found in ductal carcinoma in situ (DCIS) in one dataset (Figure 1A, Table S1). To search for the possible mechanism by which the transcriptional levels of the *ANGPT*/*ANGPTL* genes were altered in BCs, we examined the DNA copy number changes of the *ANGPT*/*ANGPTL* genes in the TCGA-BRCA database and found a frequent increase in *ANGPT1*, *ANGPT4*, and *ANGPTL1* and a frequent decrease in *ANGPT2*, *ANGPTL5,* and *ANGPTL7* in BCs (Figure 2, left panel). Surprisingly, we discovered that the transcriptional expression levels were not significantly associated with their copy numbers, except in the case of *ANGPTL3* (Figure 2, right panel). These findings indicate that DNA copy number changes do not contribute to the downregulation of *ANGPT*/*ANGPTL* genes, suggesting that their expression is mainly controlled by other mechanisms such as methylation and the regulation of transcriptional factors.

### 3.2. Prognostic Impact of ANGPT/ANGPTL Genes in Breast Cancer Patients

**Figure 3:**
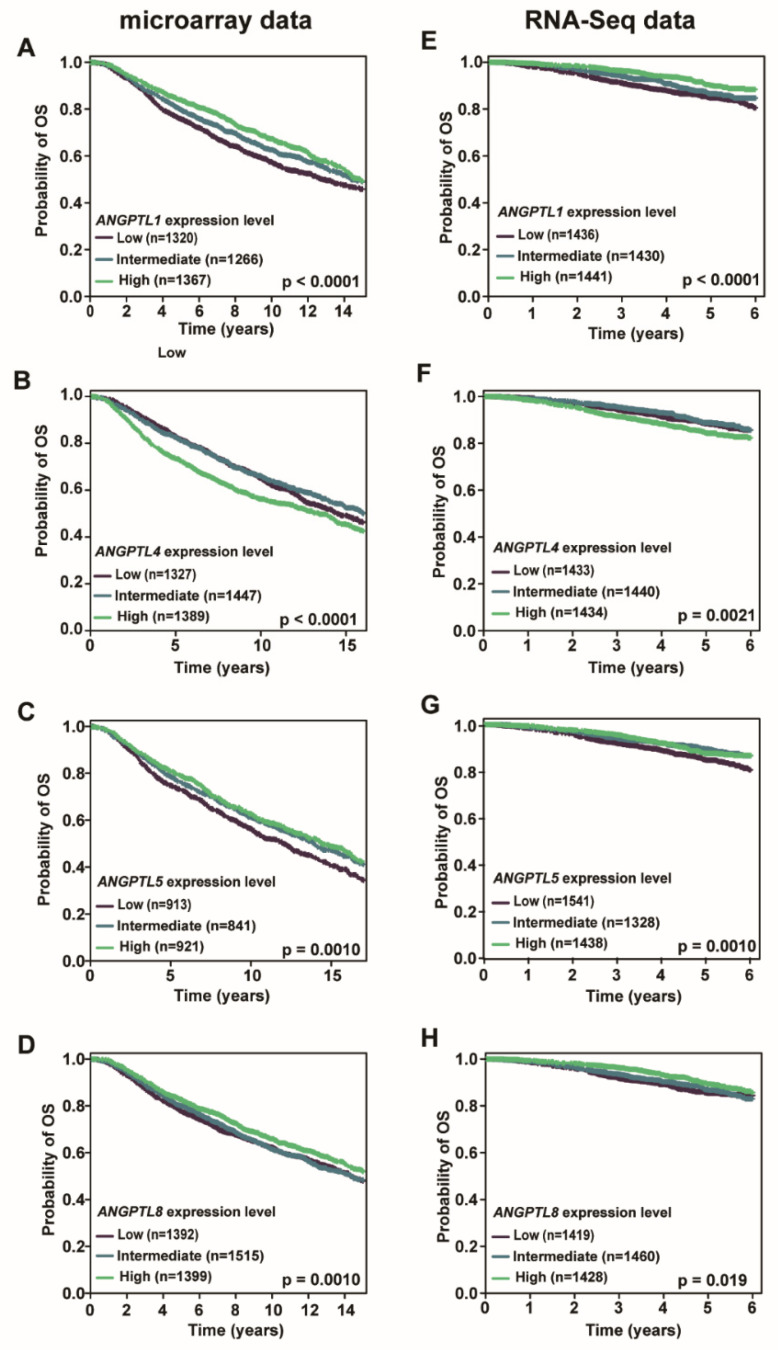
Association between *ANGPT*/*ANGPTL* genes and overall survival (OS) in breast cancer patients. Transcriptional levels of *ANGPTL1*, *4*, *5*, and *8* are significantly associated with OS in BC patients in both microarray (**A**–**D**) and RNA-seq (**E**–**H**) data. (**A**,**E**) *ANGPTL1*. (**B**,**F**) *ANGPTL4.* (**C**,**G**) *ANGPTL5.* (**D**,**H**) *ANGPTL8*.

To investigate whether transcriptional levels of individual *ANGPT*/*ANGPTL* genes were associated with OS, we conducted a meta-analysis using bc-GenExMiner v4.6. A meta-analysis of microarray data revealed that the expression levels of *ANGPTL1*, *5*, and *8* positively correlated with the OS, while the expression levels of *ANGPT2* and *ANGPTL4* negatively correlated with the OS in BC patients (*p* < 0.05, Figure 3, Figure S1). Using RNA-seq data, we found that the expression levels of *ANGPT4* and *ANGPTL1*, *5*, *7*, and *8* positively correlated with the OS, while the expression levels of *ANGPTL4* negatively correlated with the OS in BC patients (*p* < 0.05, Figure 3, Figure S1). These findings indicated that only *ANGPTL1*, *4*, *5*, and *8* are consistently associated with OS in both the microarray and RNA-seq data.

**Figure 4:**
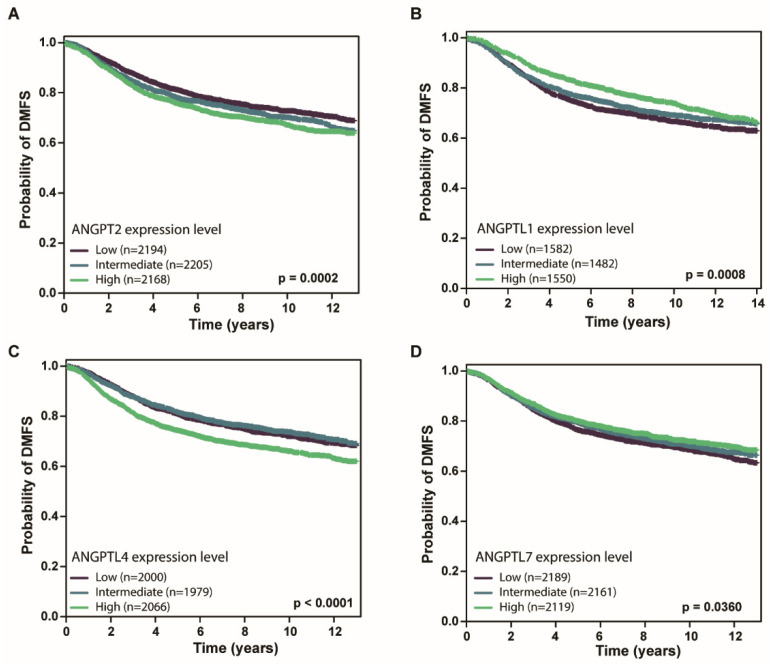
Association between *ANGPT*/*ANGPTL* genes and DMFS in breast cancers. Transcriptional levels of *ANGPT2* (**A**), *ANGPTL1* (**B**), *ANGPTL4* (**C**), and *ANGPTL7* (**D**) are significantly associated with distant metastasis-free survival (DMFS) in BC patients.

Some studies report that some of the *ANGPT*/*ANGPTL* genes play a critical role in tumor progression [12,15]. Next, we assessed the association between *ANGPT*/*ANGPTL* genes and DMFS using bc-GenExMiner, where some microarray studies contain the DMFS information. A meta-analysis showed that *ANGPTL1* and 7 were positively correlated with the DMFS, while the expression levels of *ANGPT2* and *ANGPTL4* were negatively correlated with the DMFS (Figure 4, Figure S2). All these findings support the evidence that some *ANGPT*/*ANGPTL* genes have a prognostic impact in BCs.

### 3.3. Molecular-Subtype-Dependent Prognostic Impact of ANGPT/ANGPTL Genes in Breast Cancers

**Figure 5:**
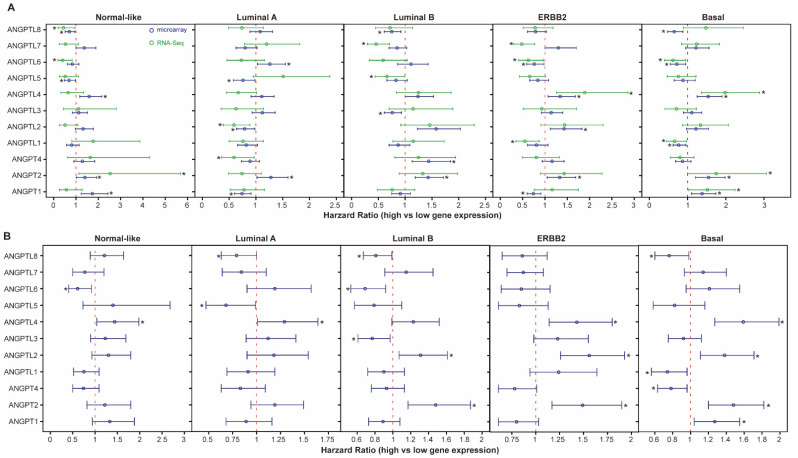
Prognostic impact of *ANGPT*/*ANGPTL* genes in different molecular subtypes. (**A**) Association between *ANGPT*/*ANGPTL* genes and OS in each molecular subtype. (**B**) Association between *ANGPT*/*ANGPTL* genes and DMFS in each molecular subtype. Open circles indicate hazard ratio (HR) and bars represent 95% confidence interval (CI) of HR. * Indicates *p* < 0.05. HR, 95% CI, with *p*-values obtained from univariate Cox regression analysis.

The molecular subtype is an important prognostic factor in BCs. Therefore, we examined whether stratifying tumors according to their molecular subtype could reveal additional information about the association between ANGPT/ANGPTL genes and BCs. First, each patient was assigned to a molecular subtype based on PAM50 [31]. The frequencies of the copy number changes in ANGPT/ANGPTL genes were found to be significantly different in different molecular subtypes (Figure S3). We then performed an impact analysis of ANGPT/ANGPTL genes on the OS and DMFS of patients in each molecular subtype and found that the association between ANGPT/ANGPTL genes and the OS and DMFS strongly depended on the molecular subtype (Figure 5). For example, significant association between transcriptional levels of ANGPTL1 and the OS and DMFS was only found in the basal type (Figure 5).

**Figure 6:**
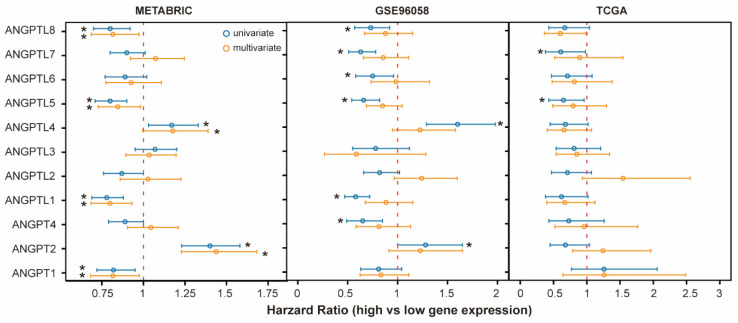
Association between *ANGPT*/*ANGPTL* genes and OS in different datasets. Open circles indicate hazard ratio (HR) and bars represent 95% confidence interval (CI) of HR. * Indicates *p* < 0.05. HR, 95% CI, with *p*-values obtained from univariate Cox regression analysis (blue circles and bars) or multivariate Cox regression analysis including clinical factors (age, tumor size, stage, and ER and PR status) and molecular subtypes (yellow circles and bars).

To assess the prognostic impact of ANGPT/ANGPTL genes independently of clinical factors and molecular subtypes, we checked all available datasets and only found three datasets that contained both the transcriptional data of all ANGPT/ANGPTL genes and data on clinical factors. Consistently with the findings of the meta-analysis described above, some ANGPT/ANGPTL genes showed significant association with the OS according to univariate Cox regression (Figure 6). However, multivariate Cox regression analyses (including age, pathological stage, ER status, PR status, tumor size, and molecular subtype) were only significant in one dataset after adjusting for clinical factors and molecular subtypes (Figure 6).

Taken together, our findings suggest that the prognostic impacts of ANGPT/ANGPTL genes are remarkably dependent on clinical factors and molecular subtypes.

### 3.4. Biological Functions of ANGPT/ANGPTL Genes in Breast Cancers Elucidated via Gene Co-Expression Network

**Figure 7:**
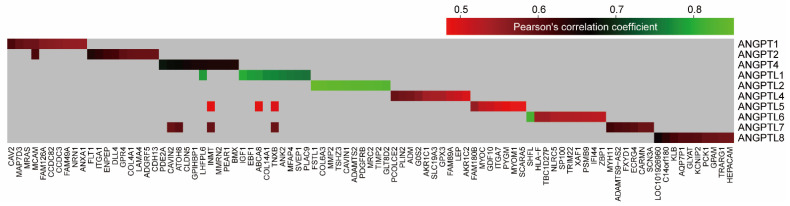
Top 10 genes that are positively correlated to each ANGPT/ANGPTL gene. For a complete list, refer to Table S2.

**Figure 8:**
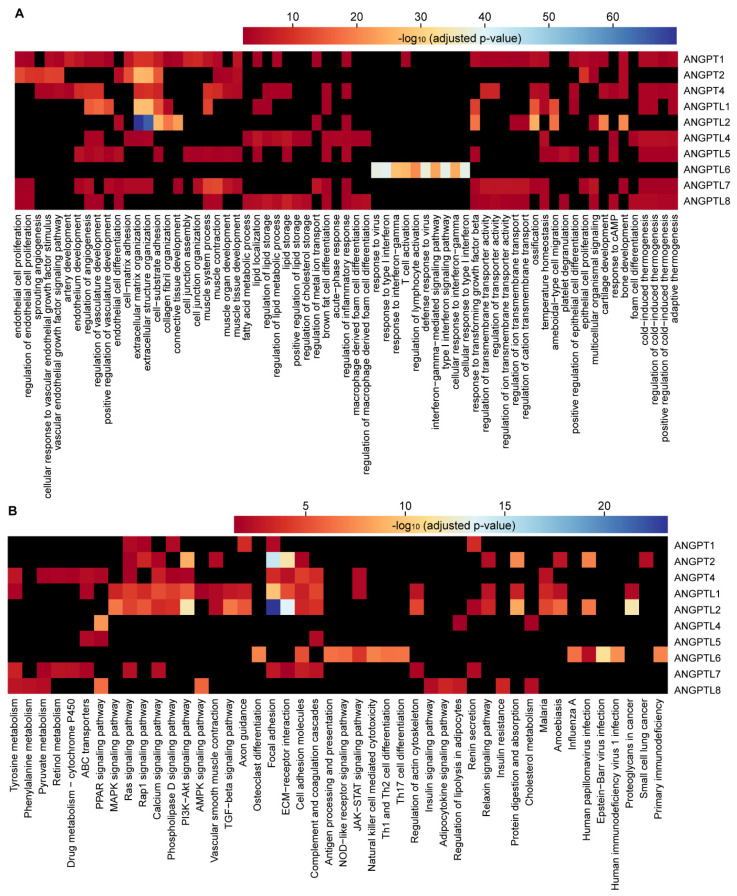
Elucidation of biological functions for *ANGPT*/*ANGPTL* genes using gene co-expression networks. (**A**) Heatmap presentation of top ten biological processes identified by GO functional enrichment analysis of the genes positively co-expressed with *ANGPT*/*ANGPTL* genes. (**B**) Heatmap presentation of top ten pathways identified by KEGG analysis of the genes positively co-expressed with *ANGPT*/*ANGPTL* genes. The cutoff for significance is adjusted *p* < 0.05. Black squares indicate no significance.

Although many studies have revealed various functions of *ANGPT*/*ANGPTL* genes [14,15], to obtain further insight into their differences with respect to the underlying mechanisms of tumor development and progression a co-expression analysis of individual *ANGPT*/*ANGPTL* genes was performed for the RNA-seq data using bc-GenExMiner. A number of genes that are significantly co-expressed with *ANGPT*/*ANGPTL* genes are shown in Table 2 (|r| ≥ 0.40; *p* < 1.00 × 10^−4^). Distinct sets of the genes were co-expressed with *ANGPT*/*ANGPTL* genes (Figure 7, Table S2). Gene Ontology (GO) functional enrichment analysis of these co-expressed genes showed significant enrichment for the distinct biological processes involved for individual *ANGPT*/*ANGPTL* genes (adjusted *p*-value < 0.05, Figure 8A, Figure S4, Table S3). Not surprisingly, it was found that the genes that were positively correlated with *ANGPT1*, *2*, and *4* and *ANGPTL1* and *5* were significantly enriched for the biological processes involved in angiogenesis (Figure 8A). This analysis also revealed that *ANGPT1*, *2*, and *4* and *ANGPTL1* and *2* are possibly involved in regulating the extracellular matrix (ECM), *ANGPTL6* possibly has a function in the regulation of immunity, and ANGPTL4 and 8 possibly regulate lipid metabolism (Figure 8A). Additionally, those genes negatively correlated with *ANGPT4* and *ANGPTL1* were significantly enriched for biological processes involved in the cell cycle (Figure S3). Moreover, KEGG analysis indicated that the co-expressed genes were significantly enriched for the distinct pathways involved by *ANGPT*/*ANGPTL* genes (Figure 8B, Figure S5, Table S4). These findings indicate distinct molecular mechanisms associated with *ANGPT*/*ANGPTL* genes in breast tumor development and progression.

### 3.5. Correlation of ANGPT/ANGPTL Genes with Biological Factors in the Tumor Microenvironment of Breast Cancers

The tumor microenvironment, which contains infiltrating host cells, secreted factors, and extracellular matrix proteins, profoundly influences tumor progression and therapeutic responses [32]. Therefore, finally, we assessed the correlations between *ANGPT*/*ANGPTL* genes and biological factors in the tumor microenvironment of breast cancers using TCGA data (Table S5). Consistently with the biological function enrichment analysis of the co-expressed genes, ANGPTL6 was strongly and significantly correlated with the immune score (Table 3). Except for ANGPTL3, the remaining *ANGPT*/*ANGPTL* genes were significantly correlated with the stroma and microenvironment scores, cancer-associated fibroblasts, and endothelial cells (Table 3). These findings suggest that the contribution of *ANGPT*/*ANGPTL* genes to BC development and progression may be through the regulation of microenvironments.

## 4. Discussion

It is well known that tumor metastasis is the real culprit and underlying cause of most BC-related deaths [1]. It is urgently necessary to design and develop effective therapeutics to block metastases. In this study, we used multiple bioinformatics tools to delineate the potential roles of 11 *ANGPT*/*ANGPTL* genes in BC since few of them have been well studied. However, ANGPT2 has been shown to play an important role in BC in many studies [33,34,35,36,37]. The ANGPT/ANGPTL proteins play a critical role in the regulation of cancer angiogenesis, which is an essential process for tumor metastasis [6,8,9]. Summarizing our findings, we conclude that ANGTPL1 and 4 are the most promising potential targets with respect to BC, although further investigations are still needed, as we discuss in detail below.

We robustly observed that ANGTPL1 and 4 were significantly downregulated in BCs, and their expression levels were significantly associated with the OS and DMFS of patients. In contrast, for the others, significance was only found in a subset of the data. One study showed that ANGPTL1 inhibits BC cell migration and invasion in vitro [16]. It is worth noting that transcriptome profiling of metastatic canine mammary carcinomas shows the significant downregulation of ANGPT2 and ANGPTL1-4 compared to normal mammary glands [38]. Consistently with these results, our study shows that high expression levels of ANGPTL1 significantly prolong the DMFS of BC patients. The co-expression network and function enrichment analysis revealed that in addition to the regulation of angiogenesis as a key essential anti-angiogenic protein [13], ANGPTL1 affects ECM regulation and suppresses cell cycles. These results suggest that ANPTL1 plays a tumor-suppressive role in BC. Studies of other cancer types support these results. It has been reported that the *ANGPTL1* transcript is downregulated in lung, prostate, kidney, thyroid, and urinary bladder cancer [39], and that ANGPTL1 suppresses metastasis in hepatocellular carcinoma [17,18], colorectal cancer [19,20,21], and lung cancer [16]. Therefore, ANGPTL1 acts as a general tumor suppressor gene in human cancers.

In addition to angiogenesis, ANGPTL4 has been reported to be involved in the regulation of lipoprotein metabolism [40]. We demonstrated that *ANGPTL4* is co-expressed with well-known genes involved in lipid metabolism. Moreover, many studies have reported the involvement of ANGPTL4 in BCs. ANGPTL4 is transcriptionally regulated by TGFβ and serves as an important mediator for TGFβ1 to prime BCs for lung metastasis [41] and TGFβ2-induced BC brain metastasis [42]. The depletion of ANGPTL4 inhibits obesity-induced angiogenesis and tumor growth [43]. Consistently with these reports, we found that a high level of ANGPTL4 significantly shortens the DMFS of BC patients. One study showed that ANPTL4 is an independent poor prognostic factor for the OS and disease-free survival (DFS) of BC patients [44]. We also observed that high levels of ANGPTL4 significantly shorten the OS of BC patients. It is worth noting that there are contradictory data in the literature about its expression alteration and its functions in human cancers. For example, a recent study demonstrated that ANGPTL4 inhibits cell migration and that high levels of ANGPTL4 prolong the OS and DFS of patients with triple-negative BC [45]. However, many studies of other types of cancer suggest that ANGPTL4 functions as an oncogene [46,47,48]. It is possible that these discrepancies are due to alternative splicing of ANGPTL4. In addition, these contradictory findings suggest a multifaceted role for ANGPTL4 in human cancers. Therefore, further investigation is required into *ANGPTL4* regulatory circuits and the definition of specific molecular events that mediate its various biological functions in different cancer stages.

A limitation of this study is that all conclusions were based on bioinformatics analyses, which require to be verified by experimental and clinical studies. Nevertheless, our study uncovered the importance of *ANGPT*/*ANGPTL* genes in BC development and progression and can guide future research.

## 5. Conclusions

Our findings provide strong evidence for the distinct clinical impacts and biological functions of ANGPT/ANGPTL proteins in BC development and progression, suggesting that some of them, such as ANGPTL1 and 4, could be potential therapeutic targets for BCs.

## Figures and Tables

**Table 1:** Mutation frequencies of *ANGPT*/*ANGPTL* genes in breast cancers.

Gene Name	TCGA (%)	COSMIC (%)
*ANGPT1*	0.9	4.42
*ANGPT2*	0.3	1.59
*ANGPT4*	1.0	2.29
*ANGPTL1*	0.6	1.05
*ANGPTL2*	0.0	0.93
*ANGPTL3*	0.5	0.35
*ANGPTL4*	0.2	0.70
*ANGPTL5*	0.4	1.16
*ANGPTL6*	0.1	0.81
*ANGPTL7*	0.1	0.39
*ANGPTL8*	0.1	0.27

**Table 2:** The number of genes significantly co-expressed with *ANGPT*/*ANGPTL* genes in breast cancers.

Gene Name	Positive Correlation	Negative Correlation
*ANGPT1*	483	14
*ANGPT2*	209	0
*ANGPT4*	722	92
*ANGPTL1*	1255	185
*ANGPTL2*	1245	30
*ANGPTL3*	0	0
*ANGPTL4*	104	0
*ANGPTL5*	169	0
*ANGPTL6*	263	0
*ANGPTL7*	506	0
*ANGPTL8*	113	0

**Table 3:** Correlation between the expression level of *ANGPT*/*ANGPTL* genes and biological factors in the tumor microenvironment of breast cancers.

Gene Name	Immune Score		Stroma Score		Microenvironment Score		Cancer Associated Fibroblast		Endothelial Cell	
	Rho	*p*-Value	Rho	*p*-Value	Rho	*p*-Value	Rho	*p*-Value	Rho	*p*-Value
*ANGPT1*	0.174	3.423 × 10^−8^	0.406	1.123 × 10^−40^	0.443	5.859 × 10^−49^	0.319	6.515 × 10^−25^	0.344	6.856 × 10^−29^
*ANGPT2*	−0.024	0.447	0.271	4.090 × 10^−18^	0.113	3.749 × 10^−4^	0.170	6.527 × 10^−8^	0.430	4.873 × 10^−46^
*ANGPT4*	−0.007	0.830	0.538	1.529 × 10^−75^	0.354	1.201 × 10^−30^	0.449	1.595 × 10^−50^	0.486	7.086 × 10^−60^
*ANGPTL1*	0.093	0.003	0.713	3.875 × 10^−155^	0.536	6.213 × 10^−75^	0.627	1.192 × 10^−109^	0.579	4.948 × 10^−90^
*ANGPTL2*	0.073	0.022	0.646	1.691 × 10^−118^	0.430	4.654 × 10^−46^	0.637	2.645 × 10^−114^	0.475	4.841 × 10^−57^
*ANGPTL3*	−0.024	0.458	0.015	0.635	0.006	0.856	0.018	0.566	0.020	0.524
*ANGPTL4*	0.002	0.945	0.373	4.689 × 10^−34^	0.258	1.649 × 10^−16^	0.262	5.285 × 10^−17^	0.277	5.923 × 10^−19^
*ANGPTL5*	0.049	0.124	0.394	2.990 × 10^−38^	0.303	1.452 × 10^−22^	0.326	5.901 × 10^−26^	0.324	1.146 × 10^−25^
*ANGPTL6*	0.355	7.858 × 10^−31^	0.168	9.353 × 10^−8^	0.390	1.809 × 10^−37^	0.185	4.235 × 10^−9^	0.121	1.268 × 10^−4^
*ANGPTL7*	−0.043	0.180	0.488	1.916 × 10^−60^	0.313	5.412 × 10^−24^	0.435	3.310 × 10^−47^	0.387	6.403 × 10^−37^
*ANGPTL8*	−0.100	0.002	0.440	3.182 × 10^−48^	0.224	9.426 × 10^−13^	0.316	1.822 × 10^−24^	0.372	5.360 × 10^−34^

## Data Availability

All data used in the study were downloaded from publicly available databases.

## Supplementary Materials

The following are available online at https://www.mdpi.com/article/10.3390/cells10102590/s1, Figure S1: Association of ANGPT1, 2, and 4, and ANGPTL2, 3, 6, and 7 with OS in both microarray and RNA-seq data. Figure S2: Association of ANGPT1 and 4, and ANGPTL2, 3, 6, and 8 with DMFS in microarray data. Figure S3: Heatmap presentation of top ten biological processes identified by GO functional enrichment analysis of the genes negatively co-expressed with angiopoietin/angiopoietin-like genes. Figure S4: Heatmap presentation of top ten pathways identified by KEGG analysis of the genes negatively co-expressed with angiopoietin/angiopoietin-like genes. Figure S5: Heatmap presentation of top ten pathways identified by KEGG analysis of the genes negatively co-expressed with ANGPT / ANGPTL genes. Table S1: Fold change (FC) and adjusted *p*-value (adj.P.Val)for angiopoietin/angiopoietin-like genes in different GEO datasets generated by GEO2R. Table S2: List of the genes significantly co-expressed with angiopoietin/angiopoietin-like genes in RNA-seq data. Table S3: Gene ontology (GO) functional enrichment analysis of the genes significantly co-expressed with angiopoietin/angiopoietin-like genes in RNA-seq data for biological processes. *adj.P.Val = adjusted *P*-Value. Table S4: Encyclopedia of Genes and Genomes (KEGG) pathway enrichment analysis of the genes significantly co-expressed with angiopoietin/angiopoietin-like genes in RNA-seq data. *adj.P.Val = adjusted *P*-Value. Table S5: Data of the expression of angiopoietin/angiopoietin-like genes and biological factors in tumor mi-croenvironment of breast cancers in TCGA.
