# Supplementary material for: Distinct Clinical Impact and Biological Function of Angiopoietin and Angiopoietin-like Proteins in Human Breast Cancer
Source: Cells. 2021 Sep 29;10(10):2590. doi: 10.3390/cells10102590 (PMC8534176; doi:10.3390/cells10102590)
Supplement: Supplementary file 1 [file cells-10-02590-s001.zip › FigureS4.pdf]

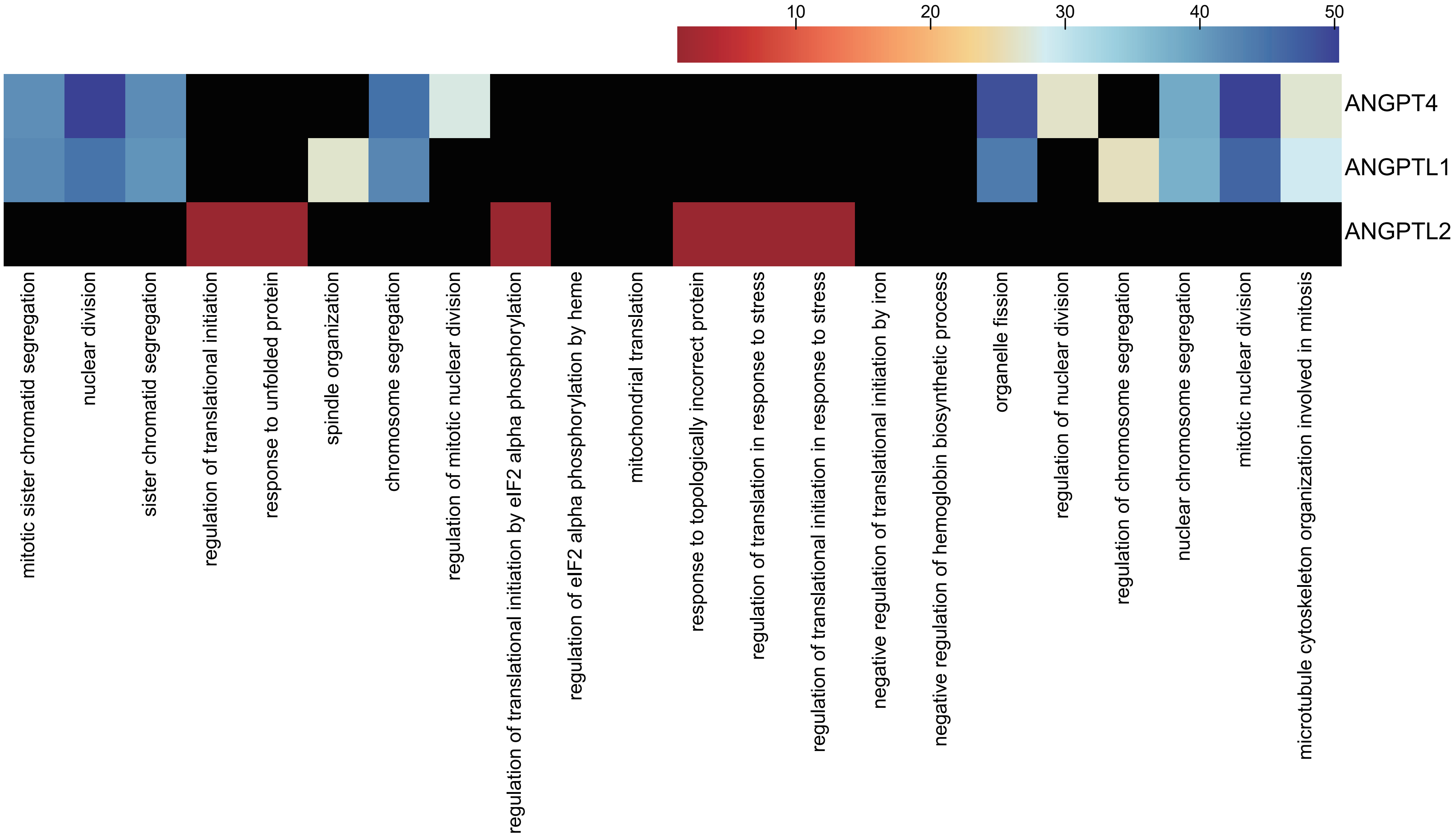

Figure S4 Heatmap presentation of top ten biological processes identified by GO functional enrichment analysis of the genes negatively co-expressed with *ANGPT / ANGPTL* genes.
