# Supplementary figures and images for: Distinct Clinical Impact and Biological Function of Angiopoietin and Angiopoietin-like Proteins in Human Breast Cancer
Source: Cells. 2021 Sep 29;10(10):2590. doi: 10.3390/cells10102590 (PMC8534176; doi:10.3390/cells10102590)

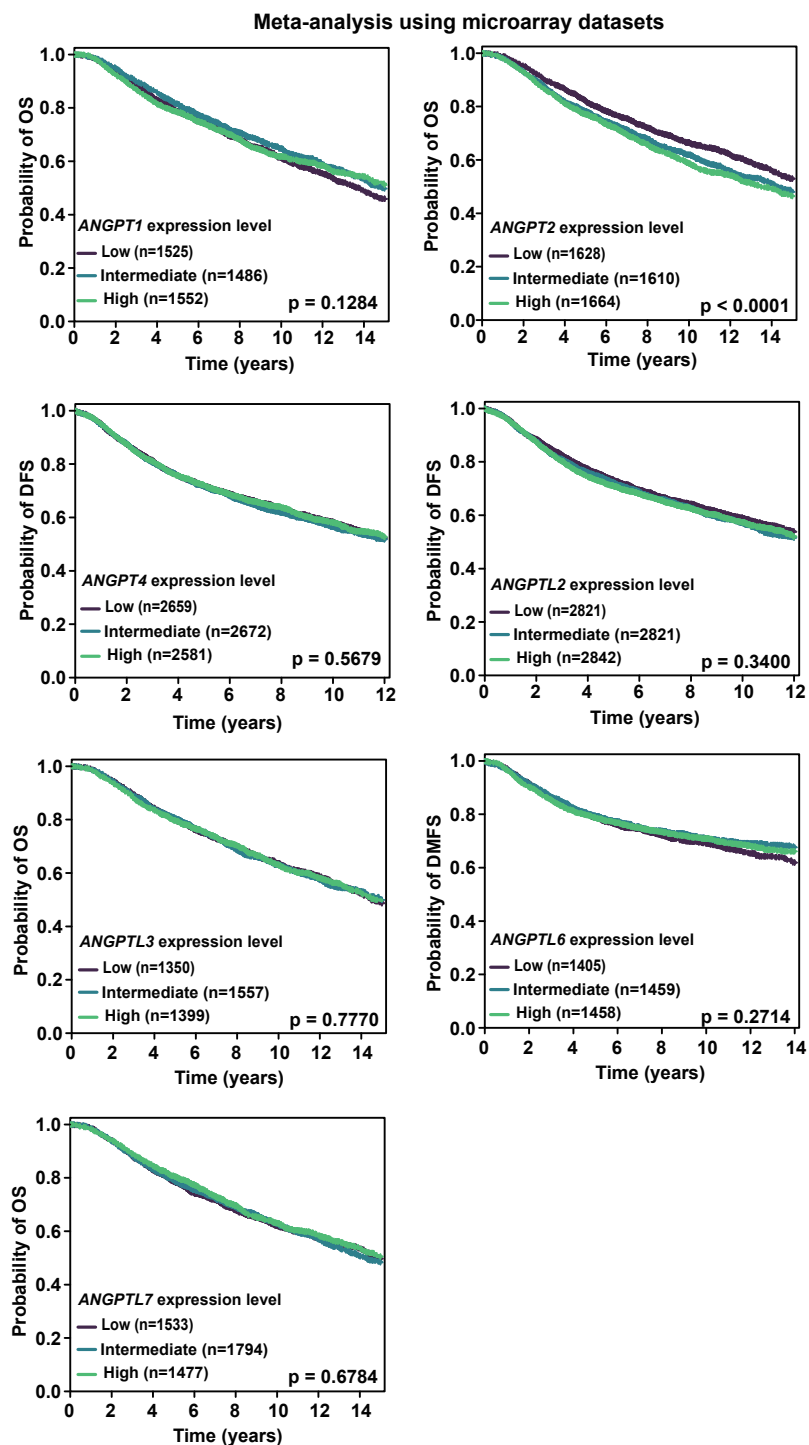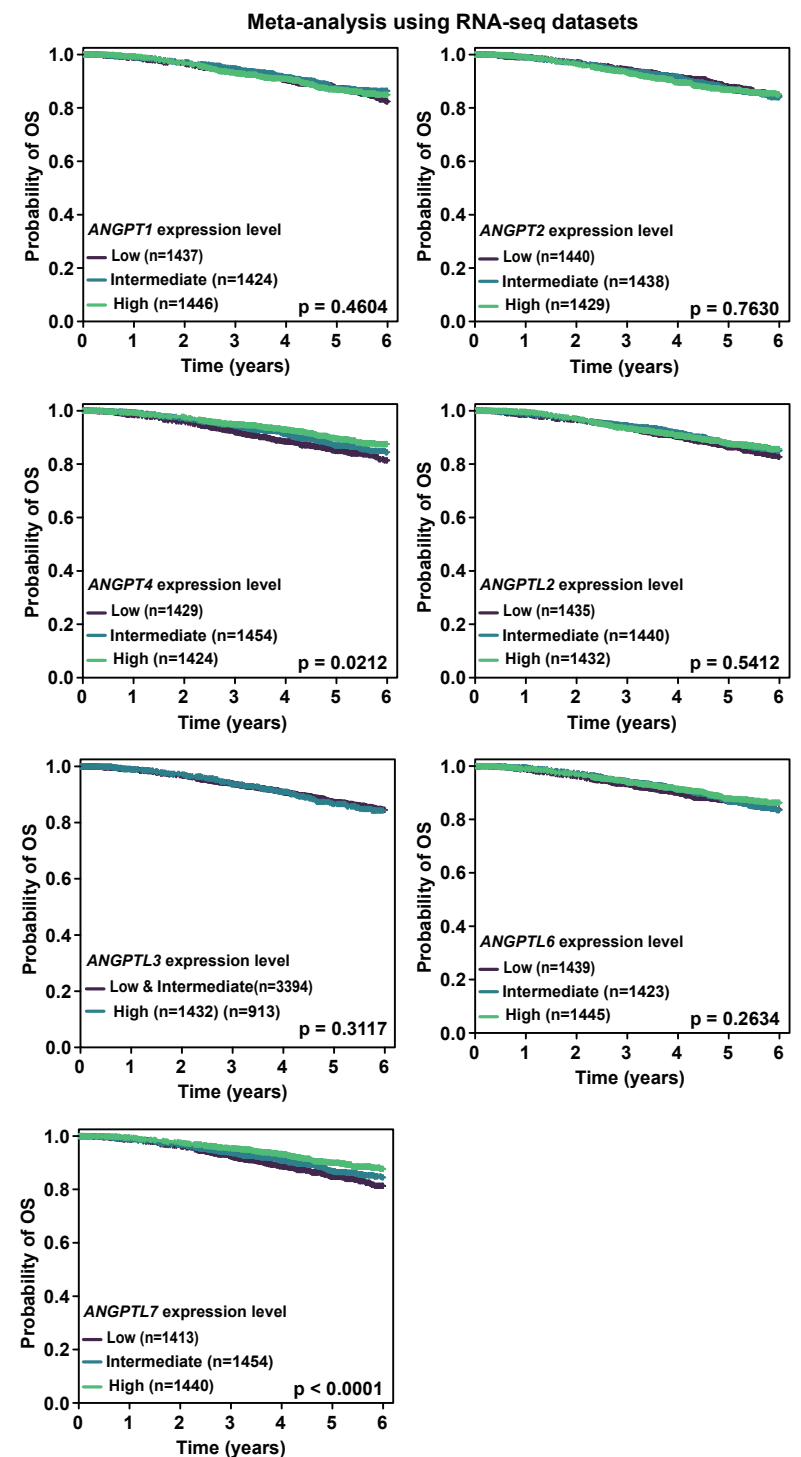

**Figure S1 Association of *ANGPT1*, *2*, and *4*, and *ANGPTL2*, *3*, *6*, and *7* with OS in both microarray and RNA-seq data.**

Supplement: Supplementary file 1 [file cells-10-02590-s001.zip › FigureS1.pdf]

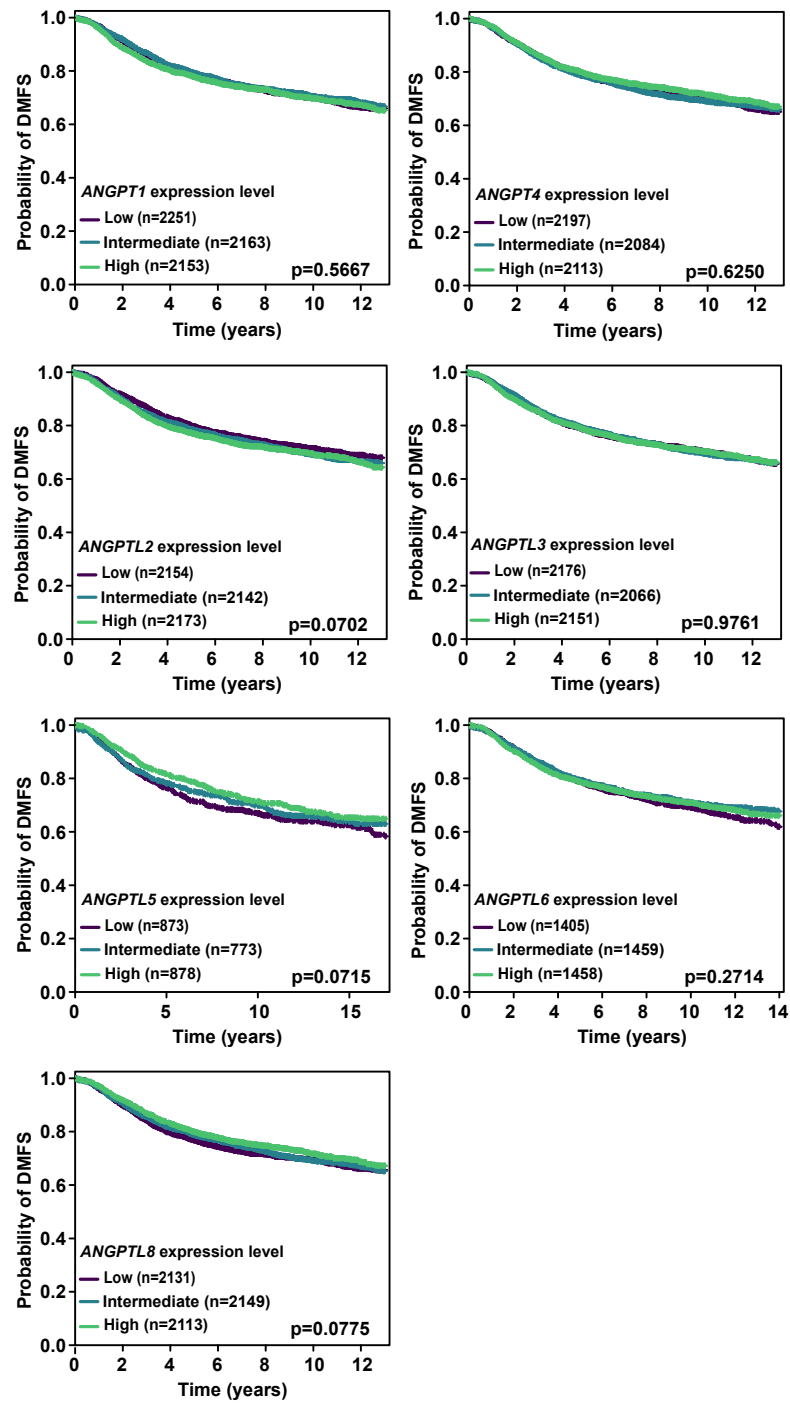

**Figure S2 Association of *ANGPT1* and 4, and *ANGPTL2*, 3, 5, 6, and 8 with DMFS in microarray data.**

Supplement: Supplementary file 1 [file cells-10-02590-s001.zip › FigureS2.pdf]
